# Scutellarein promotes diabetic wound repair and preserves fibroblast calcium-mitochondrial homeostasis

**DOI:** 10.3389/fphar.2026.1925629

**Published:** 2026-09-17

**Authors:** Yizhuo Huang, Shengzhou Shan, Mengling Chang, Yi Liu, Yucheng Yao, Jun Yang, Fei Liu

**Affiliations:** 1 Department of Plastic and Reconstructive Surgery, Shanghai Ninth People’s Hospital, Shanghai Jiao Tong University School of Medicine, Shanghai, China; 2 The Second Affiliated Hospital of Kunming Medical University, Kunming, China; 3 Department of Plastic and Reconstructive Surgery, The First People’s Hospital of Lancang Lahu Autonomous County, Lancang, Yunnan, China

**Keywords:** calcium signaling, diabetic wound, fibroblast, mitochondrial dysfunction, oxidative stress, P2RX1, scutellarein

## Abstract

**Background:**

Diabetic wounds are characterized by delayed repair associated with persistent inflammation, oxidative stress, fibroblast dysfunction, mitochondrial injury, and impaired angiogenesis. Scutellarein (SCU), a structurally defined plant-derived flavonoid metabolite, has documented antioxidant and anti-inflammatory activities; however, its role and the mechanistically relevant pathways engaged in diabetic wound repair, particularly those involving calcium-mitochondrial homeostasis and purinergic signaling, remain incompletely defined.

**Methods:**

We administered local SCU in a streptozotocin-induced diabetic mouse cutaneous wound model and, in ${\text{H}}_{2}{\text{O}}_{2}$ -injured L929 fibroblasts, assessed scratch closure, survival, redox balance, mitochondrial morphology and function, and calcium overload. We then used RNA sequencing (RNA-seq) and adenosine triphosphate (ATP) challenge/reversal experiments to examine purinergic calcium signaling. These analyses identified *P2rx1*—the gene encoding P2RX1 (P2X purinoceptor 1), an ATP-gated cation channel that mediates rapid calcium entry—as a candidate purinergic signal rather than a validated target.

**Results:**

SCU accelerated wound closure and increased re-epithelialization, Masson-positive collagen deposition, proliferative α -SMA-positive wound cells, and angiogenic indices in diabetic wounds. In injured fibroblasts, SCU increased scratch closure, reduced cell death and reactive oxygen species (ROS) accumulation, restored the superoxide dismutase (SOD)/malondialdehyde (MDA) balance, preserved mitochondrial morphology and membrane potential, and increased cellular ATP content. RNA-seq showed *P2rx1* downregulation after SCU treatment and enrichment of calcium transport, mitochondrial, inflammatory, and wound-healing pathways. ATP addition attenuated the SCU-associated reductions in calcium overload, mitochondrial depolarization, and inflammatory gene expression, consistent with a contribution from broad purinergic calcium signaling.

**Conclusion:**

In experimental models, SCU promoted diabetic wound repair and protected fibroblasts from oxidative injury. The data support preservation of calcium-mitochondrial homeostasis as a mechanistically relevant axis, and broad purinergic signaling, possibly involving P2RX1, may contribute to these effects; direct receptor-perturbation studies are warranted.

## 1 Introduction

Diabetic wounds remain a clinically important manifestation of chronic diabetic complications because hyperglycemia, persistent inflammation, microvascular dysfunction, and impaired reparative cell behavior collectively delay progression through the normal stages of tissue repair (Pawar et al., 2021; Song et al., 2025; Theocharidis et al., 2020; Guillamat-Prats, 2021; Smith and Rai, 2024). Beyond delayed wound closure, diabetic wounds reflect a broader disruption of coordinated epithelial repair, matrix remodeling, and vascular reconstruction within a persistently inflamed microenvironment (Pawar et al., 2021; Song et al., 2025; Theocharidis et al., 2020).

Among the cellular programs disrupted in diabetic wounds, fibroblast and myofibroblast dysfunction is particularly relevant because these cells support granulation tissue formation, wound contraction, extracellular matrix (ECM) deposition, and crosstalk with angiogenic compartments (Wan et al., 2021; Rai et al., 2023; Hinz, 2016). Impaired fibroblast plasticity, defective myofibroblast activity, and reduced support for angiogenesis and re-epithelialization have been linked to poor diabetic wound outcomes (Wan et al., 2021; Rai et al., 2023; Guan et al., 2021; Yan et al., 2025). These observations support fibroblast-centered repair biology as a mechanistically informative focus for diabetic wound therapy studies.

Oxidative stress is a central barrier to wound repair in diabetes. Excess reactive oxygen species (ROS) can perpetuate inflammation, damage cellular macromolecules, and impair fibroblast survival and function in chronic wounds (Wlaschek and Scharffetter-Kochanek, 2005; Wei et al., 2009; Elajaili et al., 2025). In parallel, mitochondrial injury amplifies oxidative stress and limits the energy supply required for migration, matrix synthesis, and tissue restoration (Ma et al., 2009; Wu et al., 2010; Zhou et al., 2022). Recent diabetic wound studies further indicate that correction of redox imbalance and mitochondrial dysfunction can improve fibroblast fitness and wound-healing outcomes (Tian et al., 2025; Yu et al., 2022; Zhang et al., 2025; Guo et al., 2025). Accordingly, interventions that reduce oxidative injury and preserve mitochondrial homeostasis may have reparative potential in diabetic wounds.

Purinergic signaling is a plausible upstream link between inflammation, calcium dysregulation, and mitochondrial injury. Adenosine triphosphate (ATP)-driven purinergic pathways have been implicated in wound repair and inflammatory cell trafficking (Kotwal et al., 2015). Among the ionotropic purinergic receptors, P2RX1 (encoded by P2RX1/*P2rx1*) is an ATP-gated cation channel that mediates rapid calcium entry and is characterized by fast activation and desensitization (Lalo et al., 2010; Qiang et al., 2025). Prior work indicates that P2RX1 activity influences calcium influx-dependent physiology, inflammatory responses, and tissue-injury programs across multiple biological contexts (Scott et al., 2004; Maitre et al., 2015; Wang et al., 2017; Yu et al., 2023). Although the role of fibroblast-specific P2RX1 in diabetic skin remains insufficiently defined, the available literature supports P2RX1-associated calcium signaling as a relevant link between oxidative and mitochondrial stress. Notably, P2RX1 modulation has been reported to preserve mitochondrial dynamics and alleviate tissue injury *in vivo* (Zhuang et al., 2021).

Scutellarein (SCU), a flavonoid metabolite, is relevant to this context because prior studies support anti-inflammatory, antioxidant, and fibroblast-associated activities (Pandith et al., 2013; Nazaruk and Galicka, 2014; Miao et al., 2020; Lai and Li, 2024). Prior studies of SCU have focused mainly on its antioxidant and anti-inflammatory actions, whereas its effects on calcium-mitochondrial homeostasis and purinergic signaling in diabetic wound repair have not been examined. From an ethnopharmacological perspective, scutellarein is the defining aglycone of scutellarin and is characteristic of the genus *Scutellaria*, most notably *Scutellaria baicalensis* Georgi [Lamiaceae; Scutellariae baicalensis radix; *Huang Qin*, Baikal skullcap], a traditional botanical drug whose root is used to clear heat, dry dampness, and resolve toxins and has a long record of external use for inflammatory and ulcerative skin conditions (Shen et al., 2021); it is likewise present in *Erigeron breviscapus* (Vant.) Hand.-Mazz. [Asteraceae], the source of the clinically used scutellarin preparation *Dengzhanhuasu*. The botanical names follow the Kew Medicinal Plant Names Services (MPNS) and Plants of the World Online (POWO). Consistent with the Four Pillars of Best Practice in Ethnopharmacology and the ConPhyMP guidelines for reporting plant-derived metabolites (Heinrich et al., 2022; 2020), the botanical identity, traditional context, and phytochemical characterization of the metabolite are reported accordingly. In the present study, we evaluated whether local SCU treatment promotes diabetic wound healing in a mouse model and whether its cellular effects are associated with calcium overload, mitochondrial injury, and P2RX1-related purinergic signaling in fibroblast repair responses.

## 2 Materials and methods

### 2.1 Animal model of diabetic wound healing and treatment design

A streptozotocin (STZ)-induced diabetic mouse cutaneous wound model was used to evaluate the *in vivo* wound-healing effects of scutellarein (SCU). Male C57BL/6J mice aged 6 weeks were used for the *in vivo* experiments. Sex was therefore defined biologically, and sex was not analyzed as an experimental variable in this study. All animal procedures were approved by the Shanghai Ninth People’s Hospital, Shanghai Jiao Tong University School of Medicine Ethics Committee (approval No. SH9H-2021-A983-SB) and were conducted in accordance with institutional and national guidelines for the care and use of laboratory animals.

Mice were obtained from Chengxi Biotechnology Co., Ltd. (China) and housed under specific pathogen-free (SPF) conditions at 20–26 ° C and 30%–70% relative humidity under a 12 h light/dark circadian rhythm. Animal handling, anesthesia, analgesia, wound monitoring, and endpoint tissue collection were conducted to minimize pain and distress.

After overnight fasting for 12 h with free access to water, mice were administered freshly prepared STZ at 150 mg/kg by intraperitoneal injection. STZ was dissolved immediately before use in cold 0.1 M sodium citrate buffer (pH 4.5). Mice with blood glucose levels >16.7 mmol/L at 72 h after STZ injection were considered diabetic.

After successful model establishment, full-thickness cutaneous wounds were generated on the back in a splinted wound model using an 8 mm diameter wound. Wounds were created under aseptic conditions using a sterile 8 mm biopsy punch after inhalational isoflurane anesthesia (3%–4% for induction and 1.5%–2% for maintenance). Buprenorphine (0.05 mg/kg, subcutaneous injection) was administered for postoperative analgesia and repeated every 12 h for 48 h when needed. After wounding, the splinted wound area was covered with a sterile transparent dressing and protected with an external adhesive barrier; animals were monitored daily for wound appearance, dressing integrity, activity, and signs of distress or infection.

Animals were allocated to a normoglycemic control group, a diabetic model group, and a diabetic plus SCU treatment group. Eligible mice were randomly allocated to groups using a computer-generated random-number table. Investigators performing wound-area, histologic, and immunofluorescence quantification were blinded to group allocation, and group codes were revealed only after all quantitative analyses had been completed.

Scutellarein (5,6,7,4’-tetrahydroxyflavone; CAS No. 529-53-3; molecular weight 286.24 g/mol), a structurally defined flavonoid metabolite, was purchased as a pure metabolite from MedChemExpress (Monmouth Junction, NJ, USA; Cat. No. HY-N0752; purity 99.39% by reversed-phase high-performance liquid chromatography, with certificate of analysis and ^1^H-NMR/MS documentation). As a commercially sourced pure metabolite used for pharmacological testing (rather than a botanical extract), the applicable ConPhyMP requirements are those for purchased pure metabolites—supplier, catalogue/CAS number, purity (%), and a chemical-characterization statement—which are reported here (Heinrich et al., 2022); a completed ConPhyMP checklist (ConPhyMP Table 1) is provided as Supplementary Material, the structure is shown in Supplementary Figure 1, and no plant material was collected or extracted for this study. SCU was dissolved in dimethyl sulfoxide (DMSO) to prepare stock solutions and diluted to the indicated working concentrations before use; the final DMSO concentration in the applied working solution was maintained at a level considered safe for topical administration.

SCU was prepared as a 200 μ M working solution and locally administered to the wound bed and periwound region, with 500 μ L applied per wound and distributed over the wound surface and the surrounding subcutaneous periwound tissue. The control and diabetic model groups received an equal volume of the vehicle (DMSO diluted in saline at the same final concentration) on the same schedule. The first administration was performed at the time of wound modeling and repeated once every 3 days until tissue collection at the designated endpoint. The 200 μ M working concentration and the every-3-day schedule were selected on the basis of the *in vitro* dose-response experiments, preliminary *in vivo* testing, and published pharmacological, pharmacokinetic, and local-delivery data for scutellarein/scutellarin (Lai and Li, 2024; Yang et al., 2014; Xia et al., 2026). Local tissue concentration, penetration depth, retention, and systemic absorption were not quantified.

Macroscopic wound healing was monitored at days 0, 3, 7, and 14. The wound-area percentage analysis included n=5 –8 animals per group, whereas histologic and immunostaining quantifications included n=3 –5 samples per group, as indicated in the figure legends. No formal sample-size calculation was performed *a priori*; group sizes were selected on the basis of effect sizes observed in comparable streptozotocin-induced diabetic wound studies in mice (Guan et al., 2021; Huang et al., 2020; Chen et al., 2026) and were considered sufficient for the primary wound-closure and histologic endpoints.

### 2.2 Gross wound imaging and wound-closure analysis

Representative gross wound photographs were acquired on days 0, 3, 7, and 14 after wounding. Wound margins were traced to generate wound outlines, and wound area percentage was quantified longitudinally.

Gross wound images were acquired with a digital camera at a fixed distance under standardized lighting and background conditions, with a metric ruler included in each image for calibration. Wound areas were quantified using ImageJ after calibration to the ruler scale. Wound area was analyzed relative to day 0.

### 2.3 Histology and morphometric analysis of epithelial repair and collagen deposition

For histologic evaluation, wound tissues were collected at days 7 and 14, fixed in 4% paraformaldehyde for 24 h, paraffin-embedded, sectioned at 5 μ m, and stained with hematoxylin and eosin (H&E) or Masson’s trichrome using commercial staining kits (Solarbio, Beijing, China) according to the manufacturer’s instructions.

H&E-stained sections were used to evaluate epithelial regeneration, and epithelial gap was quantified. Masson’s trichrome-stained sections were used to evaluate collagen deposition, and the area of collagen-positive tissue in the wound region was quantified.

The wound area and histologic outputs were quantified in ImageJ by two researchers under blinded conditions. For histologic morphometry, the wound-bed region of interest (ROI) was defined as the granulation-tissue region between the wound edges, excluding adjacent unwounded skin. Three non-adjacent sections per specimen and three representative fields per section were analyzed using the same threshold settings across groups.

### 2.4 Immunofluorescence analysis of proliferative fibroblast-like cells and angiogenesis

To assess fibroblast-like proliferative activity in wound tissue, sections were co-stained for the proliferation marker KI67 and α -smooth muscle actin ( α -SMA) with 4’,6-diamidino-2-phenylindole (DAPI) nuclear counterstaining. KI67-positive α -SMA-positive cells were quantified as a percentage of the relevant wound-cell population. To assess angiogenesis, parallel sections were stained for cluster of differentiation 31 (CD31) with DAPI, after which capillary density and relative vessel diameter were quantified. Capillary density was reported as the number of vessels per field.

Imaging was performed on a Leica confocal microscope, and representative immunofluorescence images in this assay were acquired with a 25 μ m scale bar.

KI67-positive α -SMA-positive fibroblast-like cells were defined as DAPI-positive stromal cells within the granulation tissue showing co-localized KI67 and α -SMA signal. Vessel-associated smooth muscle cells were excluded based on morphology and location around CD31-positive vascular structures.

For angiogenesis analysis, CD31-positive lumen-like or endothelial cluster structures within the granulation tissue were counted as capillaries. Capillary density and relative vessel diameter were quantified from three non-adjacent sections per specimen and three representative fields per section using ImageJ, and the mean value was used for each specimen.

### 2.5 Fibroblast culture and oxidative stress injury model

L929 mouse fibroblasts were used to establish an *in vitro* oxidative stress injury model. L929 cells were selected as a well-characterized, readily reproducible fibroblast host for the oxidative-injury model; however, they are an immortalized line and may differ from primary dermal fibroblasts in purinergic-receptor expression, and P2RX1 protein expression and function were not directly measured in L929 cells (the P2RX1-related signal derives from *P2rx1* transcript downregulation). Cells were cultured in Dulbecco’s modified Eagle’s medium (DMEM) supplemented with 10% fetal bovine serum (FBS) and 1% penicillin/streptomycin and maintained at 37 ° C in a humidified incubator containing 5% ${\text{CO}}_{2}$ . Hydrogen peroxide ( ${\text{H}}_{2}{\text{O}}_{2}$ ) was used to induce oxidative injury, and SCU dissolved in DMSO was used as the intervention. The ${\text{H}}_{2}{\text{O}}_{2}$ model recapitulates acute oxidative injury—the stress axis targeted by this study—but does not reproduce the chronic hyperglycemic milieu of diabetic wounds.

Cell viability screening by CCK-8 (Cell Counting Kit-8) assay was performed to determine working concentrations of SCU and ${\text{H}}_{2}{\text{O}}_{2}$ . Fibroblasts were exposed to graded concentrations of SCU (25–400 μ M) and of ${\text{H}}_{2}{\text{O}}_{2}$ (0.25–4 mM) for 24 h, and viability was measured at 450 nm using a Beyotime CCK-8 reagent according to the manufacturer protocol. Based on these preliminary dose-response experiments, 50 μ M SCU and 2 mM ${\text{H}}_{2}{\text{O}}_{2}$ were selected for the subsequent protection assays: 50 μ M SCU was non-toxic to uninjured fibroblasts and conferred substantial protection against ${\text{H}}_{2}{\text{O}}_{2}$ -induced viability loss, whereas 2 mM ${\text{H}}_{2}{\text{O}}_{2}$ induced a marked and reproducible reduction in viability. For these experiments, cells were pretreated with SCU for 12 h and then exposed to ${\text{H}}_{2}{\text{O}}_{2}$ for 4 h.

### 2.6 Scratch-wound closure assay

Fibroblast scratch closure was assessed using a scratch-wound assay. Confluent fibroblast monolayers were scratched and imaged at 0 and 48 h in control and SCU-treated (50 μ M) conditions. A linear wound gap was generated with a sterile 200 μ L pipette tip after the monolayer reached near confluence. Detached cells were removed by gentle washing, and images were acquired immediately (0 h) and again at 48 h. Gap closure was quantified in ImageJ as the percentage of the original wound area that had closed.

Before scratching, fibroblast monolayers were serum-starved for 12 h. Representative scratch-assay images were acquired at the predefined 0 h and 48 h time points. No mitotic inhibitor was added during the scratch-wound migration assay, so closure reflects migration with a possible proliferative contribution. The ${\text{H}}_{2}{\text{O}}_{2}$ -injured condition was omitted from this panel because ${\text{H}}_{2}{\text{O}}_{2}$ itself is a known promigratory stimulus that would confound the gap-closure readout; consequently, the scratch assay included only the SCU-alone versus control condition, and Figure 3B therefore reports only the baseline (uninjured) effect of SCU on fibroblasts and is not used as evidence of repair under injury.

### 2.7 Live/dead staining and viability analysis

Cell survival under oxidative injury was evaluated by Live/Dead staining using Calcein-AM to label live cells and propidium iodide (PI) to label dead cells. Live and dead cell numbers were quantified across control, ${\text{H}}_{2}{\text{O}}_{2}$ , and ${\text{H}}_{2}{\text{O}}_{2}$ plus SCU groups. Cells were incubated with Calcein-AM/PI working solution for 30 min at 37 ° C in the dark and then imaged by fluorescence microscopy. Live (green) and dead (red) cells were counted from representative fields.

Live/dead cell counting was performed manually from five randomly selected non-overlapping fields per well, with at least three wells analyzed per group. Quantification was performed by investigators blinded to treatment group.

The Beyotime Calcein-AM/PI kit was used according to the manufacturer’s instructions. Imaging was performed on a Leica confocal microscope. The quantitative outputs included both live/dead cell counts and relative Calcein fluorescence intensity.

### 2.8 ROS, MDA, and SOD measurements

Intracellular ROS were assessed using the 2’,7’-dichlorodihydrofluorescein diacetate (DCFH-DA) probe. Following treatment, cells were incubated with 10 μ M DCFH-DA at 37 ° C for 30 min in the dark, washed to remove excess probe, and then imaged for green fluorescence. Relative ROS levels were quantified from normalized fluorescence intensity.

The Beyotime ROS assay kit (DCFH-DA) was used according to the manufacturer’s instructions. Imaging was performed on a Leica confocal microscope.

Lipid peroxidation was assessed by measuring malondialdehyde (MDA), and antioxidant defense was assessed by measuring superoxide dismutase (SOD) activity using Beyotime assay kits. MDA content and SOD activity were normalized to total protein concentration and reported as nmol/mg protein and U/mg protein, respectively.

### 2.9 Mitochondrial morphology analysis

Mitochondrial morphology was examined by MitoTracker Red CMXRos staining under standard live-cell conditions (approximately 200 nM, 30 min at 37 ° C). Representative fluorescence images were binarized and used to calculate mean aspect ratio (elongation) and mitochondrial count (fragmentation). ImageJ was used for mitochondrial-image binarization and morphometric analysis. Imaging was performed on a Leica confocal microscope, and representative mitochondrial morphology images were presented with 1 μ m scale bars.

### 2.10 Mitochondrial membrane potential assay

Mitochondrial membrane potential was measured using the 5,5’,6,6’-tetrachloro-1,1’,3,3’-tetraethylbenzimidazolylcarbocyanine iodide (JC-1) probe. Cells were incubated with JC-1 working solution for 20 min at 37 ° C in the dark, washed, and imaged for red (J-aggregate) and green (monomer) fluorescence. The red/green fluorescence ratio was used as the quantitative metric for mitochondrial membrane potential. The Beyotime enhanced mitochondrial membrane potential assay kit (JC-1) was used according to the manufacturer’s instructions. Imaging was performed on a Leica confocal microscope, and representative JC-1 images were presented with 10 μ m scale bars.

### 2.11 Transmission electron microscopy and ATP measurement

Mitochondrial ultrastructure was examined by transmission electron microscopy (TEM). Cells were fixed in 2.5% glutaraldehyde in 0.1 M phosphate buffer (pH 7.4) at 4 ° C overnight, post-fixed with 1% osmium tetroxide, dehydrated through a graded ethanol series, embedded in epoxy resin, and cut into approximately 70 nm ultrathin sections. Sections were stained with uranyl acetate and lead citrate and imaged using a Hitachi HT7800 transmission electron microscope. At least 10 cells or 30 mitochondria were evaluated per condition from representative fields.

Cellular ATP content was measured using a Beyotime ATP assay kit and a luciferase-based bioluminescence readout on a multimode microplate reader. ATP levels shown in Figure 4H were calibrated to total protein concentration and reported as absolute ATP concentrations (nM); values were not rescaled to the control-group level.

### 2.12 RNA sequencing and bioinformatic analysis

To investigate transcriptional mechanisms associated with SCU treatment, RNA sequencing (RNA-seq) was performed on RNA isolated from the ${\text{H}}_{2}{\text{O}}_{2}$ -injured (vehicle) L929 fibroblast group and the SCU-treated L929 fibroblast group (i.e., on L929 cell RNA, not wound tissue), with three biological replicates per group ( n=3 vs. 3). Principal component analysis (PCA), differential expression analysis, gene ontology (GO) enrichment, and gene set enrichment analysis (GSEA) were used to characterize treatment-associated transcriptional changes.

Total RNA was extracted using TRIzol reagent according to the manufacturer’s protocol. RNA purity and concentration were evaluated using a NanoDrop 2000 spectrophotometer (Thermo Scientific, USA), and RNA integrity was assessed using an Agilent 2100 Bioanalyzer (Agilent Technologies, Santa Clara, CA, USA). RNA-seq libraries were constructed using the VAHTS Universal V10 RNA-seq Library Prep Kit (Premixed Version) according to the manufacturer’s instructions. Transcriptome sequencing and analysis were conducted by OE Biotech Co., Ltd. (Shanghai, China).

Libraries were sequenced on an Illumina NovaSeq 6000 platform to generate 150 bp paired-end reads, with an approximate sequencing depth of 6 Gb per sample. Raw FASTQ reads were processed using fastp to remove low-quality reads, and the resulting clean reads were mapped to the reference genome using HISAT2. Gene expression was calculated as fragments per kilobase of transcript per million mapped reads (FPKM), and read counts for each gene were obtained using HTSeq-count. Principal component analysis was performed in R to evaluate sample clustering. Differential expression analysis was performed using DESeq2, with q<0.05 and fold change >1.5 or <0.667 used to define differentially expressed genes. GO, Kyoto Encyclopedia of Genes and Genomes (KEGG), Reactome, and WikiPathways enrichment analyses were performed based on the hypergeometric distribution, and GSEA was performed using predefined gene sets ranked by differential expression. The RNA-seq dataset has been deposited in the NCBI Gene Expression Omnibus (GEO) under accession number GSE333609.

### 2.13 Intracellular calcium imaging and ATP reversal experiments

Intracellular calcium levels were assessed in control, ${\text{H}}_{2}{\text{O}}_{2}$ , ${\text{H}}_{2}{\text{O}}_{2}$ plus SCU, and ${\text{H}}_{2}{\text{O}}_{2}$ plus SCU plus ATP groups. Relative calcium levels were quantified from fluorescence imaging across groups to determine whether ATP addition could counteract the calcium-lowering effect associated with SCU treatment. All experiments used L929 fibroblasts.

A Beyotime calcium indicator dye from the Fluo-4 series, Fluo-4 AM (S1056), was used for live-cell calcium imaging. Cells were loaded with the dye at 37 ° C for 30 min according to the manufacturer protocol, washed, and imaged on a Leica confocal microscope.

ATP (adenosine 5’-triphosphate disodium; 100 μ M) was used as a purinergic challenge/reversal stimulus in the SCU-treated injury model. ATP (100 μ M) was added to the SCU-plus-injury group to determine whether purinergic calcium influx could attenuate the SCU-associated effects on intracellular calcium homeostasis and downstream mitochondrial function. At this concentration ATP engages multiple P2X and P2Y subtypes expressed by fibroblasts and therefore represents a broad purinergic challenge rather than a P2RX1-specific stimulus.

### 2.14 RT-qPCR validation

Reverse transcription-quantitative PCR (RT-qPCR) was used to validate expression changes in *P2rx1*, *Bcl2*, *Tnf*, and *Il1b* across experimental groups using RNA from L929 fibroblasts. *Gapdh* was used as the internal reference gene. Relative expression was calculated using the $2^{-\Delta \Delta Ct}$ method and normalized to *Gapdh*. The RT-qPCR primer sequences are listed in Supplementary Table 1.

Total RNA was extracted using TRIzol reagent, and cDNA was synthesized using a PrimeScript RT reagent kit. qPCR was performed using SYBR Green chemistry on a QuantStudio five real-time PCR system.

### 2.15 Statistical analysis

Data are presented as mean ± standard error of the mean (SEM). *In vitro* assays were performed in at least three independent experiments. Statistical significance is indicated as ns (not significant), *p<0.05 , *∗p<0.01 , *∗∗p<0.001 , and *∗∗∗p<0.0001 . Group comparisons were analyzed using analysis of variance (ANOVA). One-way ANOVA followed by Tukey’s *post hoc* test was used for endpoint comparisons among treatment groups, and repeated-measures or two-way ANOVA followed by multiple-comparison testing was used for longitudinal or time-course datasets where appropriate.

For wound-tissue datasets, n=5 -8 was used for wound-area analysis and n=3 -5 was used for histologic and immunostaining quantifications, as indicated in the figure legends. Statistical analysis was performed using GraphPad Prism. ANOVA-based analyses were followed by Tukey’s *post hoc* test for multiple comparisons unless otherwise indicated.

## 3 Results

The results are organized from tissue-level diabetic wound repair to fibroblast-centered protection against oxidative injury, followed by transcriptomic analysis and ATP reversal experiments examining purinergic/P2RX1-associated calcium-mitochondrial signaling.

### 3.1 Scutellarein accelerates diabetic wound closure and promotes epithelial and matrix repair

To assess the *in vivo* effects of SCU on diabetic wound healing, we used a STZ-induced diabetic mouse cutaneous wound model and monitored wound repair longitudinally. Representative gross wound images (Figure 1A) and traced wound outlines at the final time point (day 14; Figure 1B) indicated that the diabetic (DM) group retained larger residual wound areas than normoglycemic controls, particularly at later repair stages. Quantification of wound area percentage (Figure 1C; n=5 -8 per group) showed that the divergence between control and diabetic wounds was most evident on days 7 and 14. SCU-treated diabetic wounds had smaller residual wound areas than untreated diabetic wounds, indicating accelerated repair kinetics in this experimental model. No wound infections and no unexpected adverse events were observed in any group during the 14-day monitoring period.

**FIGURE 1:**
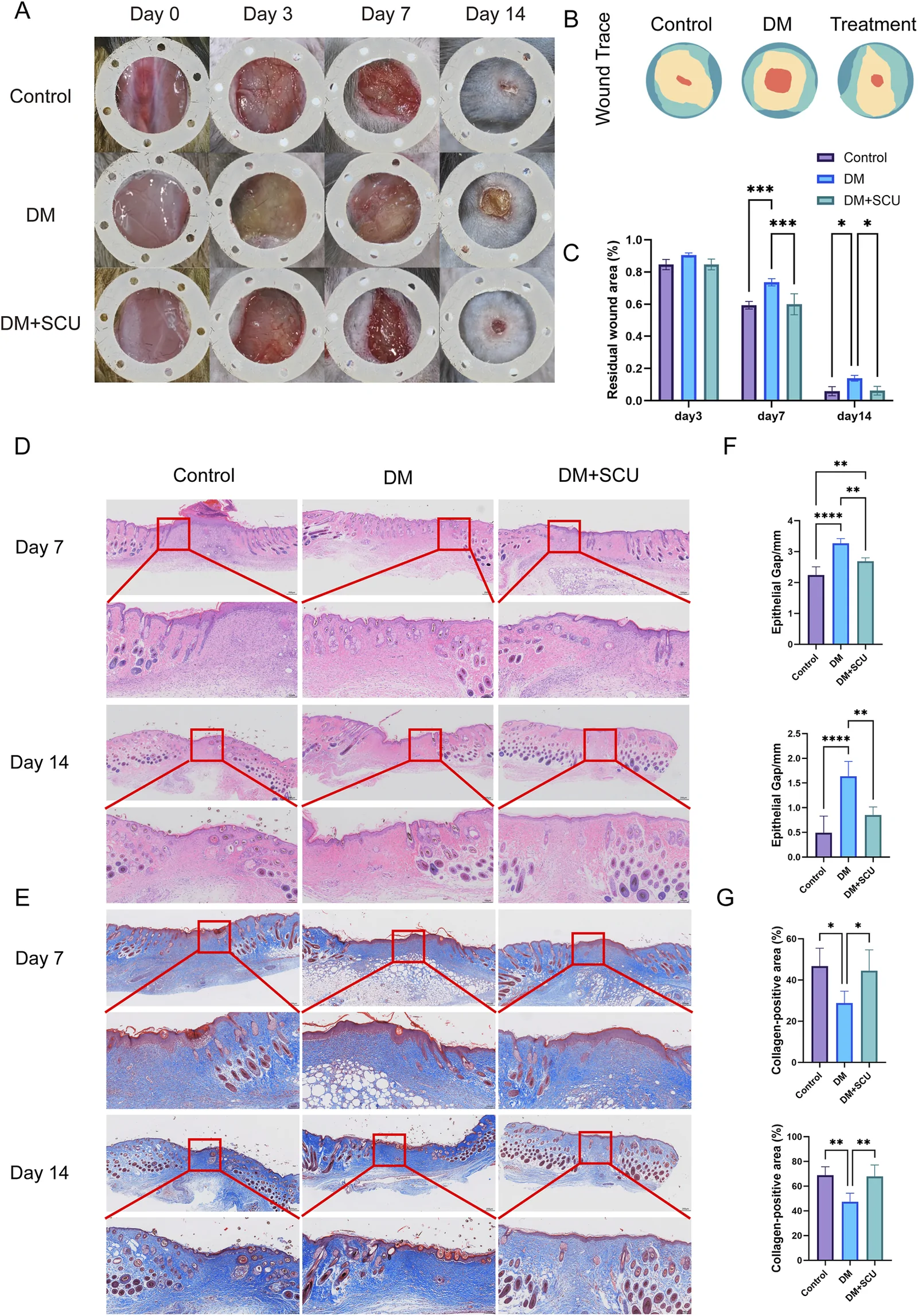
SCU promotes diabetic wound healing. Representative macroscopic wound images of control, diabetic (DM), and SCU-treated diabetic mice were captured on days 0, 3, 7, and 14 after injury **(A)** with corresponding wound-outline schematics **(B)**. Wound area percentage was quantified at days 3, 7, and 14 **(C)** n=5 -8. H&E staining of wound tissue collected on days 7 and 14 was used to evaluate epithelial regeneration **(D)** and epithelial gap was quantified **(F)** n=3 -5. Masson’s trichrome staining was used to assess collagen deposition in the wound bed at the same time points **(E)** and Masson-positive collagen area was quantified **(G)** n=3 -5. Wound-area time-course data were analyzed by two-way ANOVA with multiple-comparison testing; histologic endpoints were analyzed by one-way ANOVA with Tukey’s *post hoc* test. Data are presented as mean ± SEM. ns, not significant; *p<0.05 , **p<0.01 , ***p<0.001 , ****p<0.0001 .

Histologic examination further indicated that the effect of SCU extended beyond gross wound closure. H&E staining at days 7 and 14 (Figure 1D) showed that diabetic wounds retained a more prominent epithelial discontinuity, whereas SCU-treated wounds displayed a more continuous epithelial layer. Consistently, epithelial-gap analysis (Figure 1F; n=3 -5) showed a larger epithelial defect in the DM group and a significant reduction after SCU treatment. Thus, accelerated wound closure was accompanied by enhanced re-epithelialization.

Extracellular matrix reconstruction showed a similar pattern. Masson’s trichrome staining (Figure 1E) indicated that collagen deposition in the diabetic wound bed was reduced relative to the control group. Quantification of Masson-positive collagen area (Figure 1G; n=3 -5) supported this matrix deficit and showed significant restoration after SCU treatment. Collectively, Figure 1 indicates that SCU promotes diabetic wound repair across closure kinetics, epithelial restoration, and matrix deposition.

### 3.2 Scutellarein increases proliferative α -SMA-positive wound cells and angiogenesis in diabetic wounds

Because wound closure and matrix remodeling depend on an active granulation program, we next examined whether SCU influenced proliferative α -SMA-positive wound cells and angiogenesis within diabetic wounds. Immunofluorescence co-staining for KI67 and α -SMA (Figure 2A) was used to assess proliferative α -SMA-positive stromal cells, and quantification of KI67/ α -SMA double-positive cells (Figure 2C; n=3 -5) showed a pronounced reduction in the DM group relative to controls. SCU treatment increased this readout, suggesting partial restoration of a proliferative fibroblast-like/myofibroblast-like wound-cell population.

**FIGURE 2:**
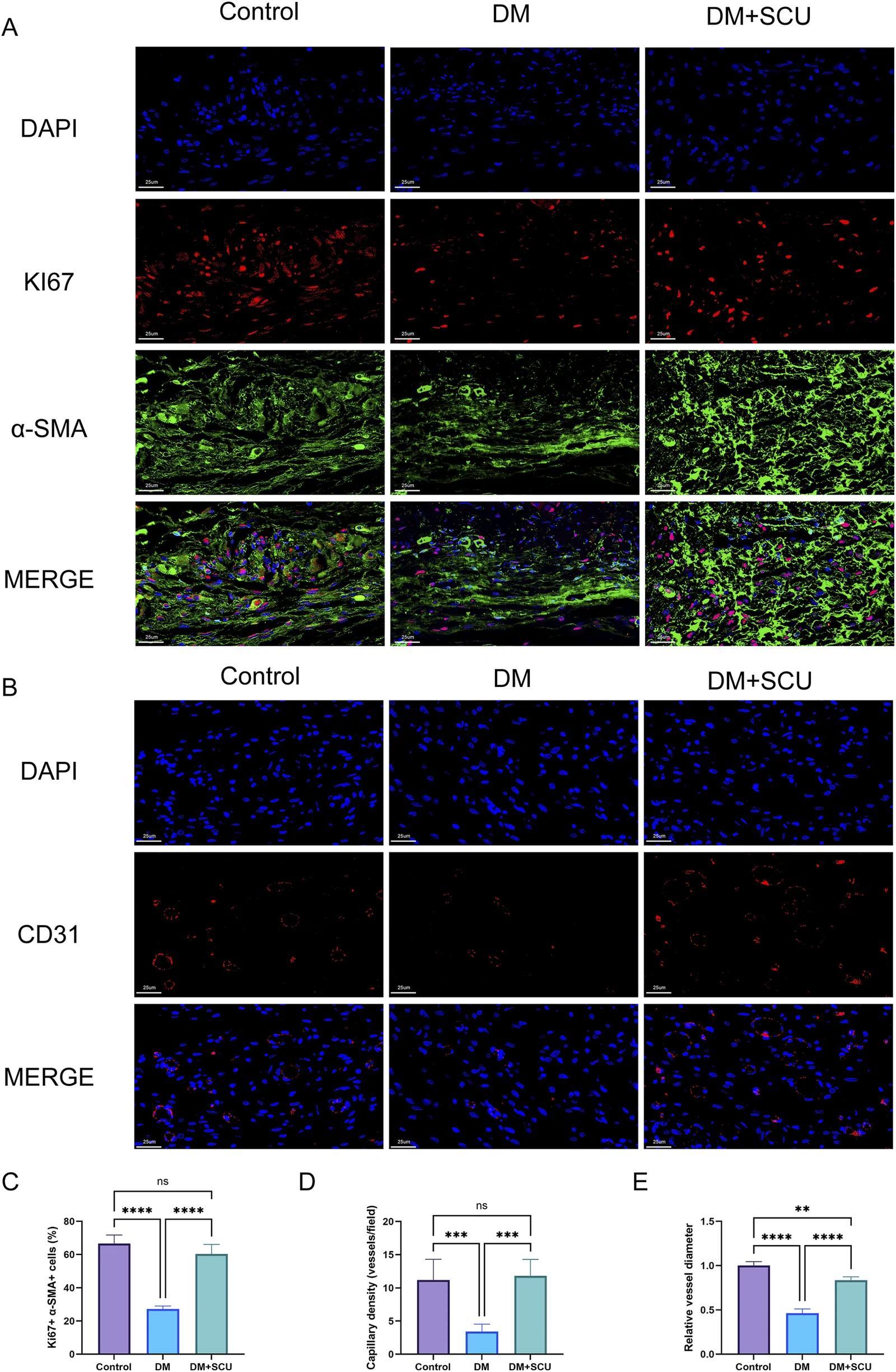
SCU increases proliferative α -SMA-positive wound cells and angiogenesis in diabetic wounds. Representative immunofluorescence images of KI67 (red), α -SMA (green), and DAPI (blue) staining in wound tissue are shown in **(A)** with quantification of KI67-positive α -SMA-positive cells in (**(C)** n=3 -5). Representative CD31 (red) and DAPI (blue) staining images are shown in **(B)** Capillary density and relative vessel diameter were quantified in **(D,E)** respectively ( n=3 -5). Scale bars, 25 μ m. One-way ANOVA with Tukey’s *post hoc* test was used for between-group comparisons. Data are presented as mean ± SEM. ns, not significant; **p<0.01 , ***p<0.001 , ****p<0.0001 .

The vascular component of granulation tissue was affected in parallel. CD31 immunofluorescence (Figure 2B) revealed reduced vascular signal in diabetic wounds, and quantification showed significant decreases in both capillary density (Figure 2D) and relative vessel diameter (Figure 2E; n=3 -5). SCU treatment increased both measures, with capillary density no longer significantly different from the control group. Viewed together with Figure 1, these results suggest that the SCU-associated increases in closure, epithelial repair, and collagen deposition occurred alongside broader restoration of the granulation-phase repair program.

### 3.3 Scutellarein enhances fibroblast scratch closure and protects against hydrogen peroxide-induced oxidative injury

To examine the fibroblast-centered cellular actions of SCU, we established an ${\text{H}}_{2}{\text{O}}_{2}$ injury model *in vitro*. Initial CCK-8 screening (Figure 3A) was used to evaluate the effects of graded SCU and ${\text{H}}_{2}{\text{O}}_{2}$ exposures on fibroblast viability and to determine working concentrations for downstream rescue experiments. Based on these screening results, 50 μ M SCU and 2 mM ${\text{H}}_{2}{\text{O}}_{2}$ were selected for subsequent assays.

**FIGURE 3:**
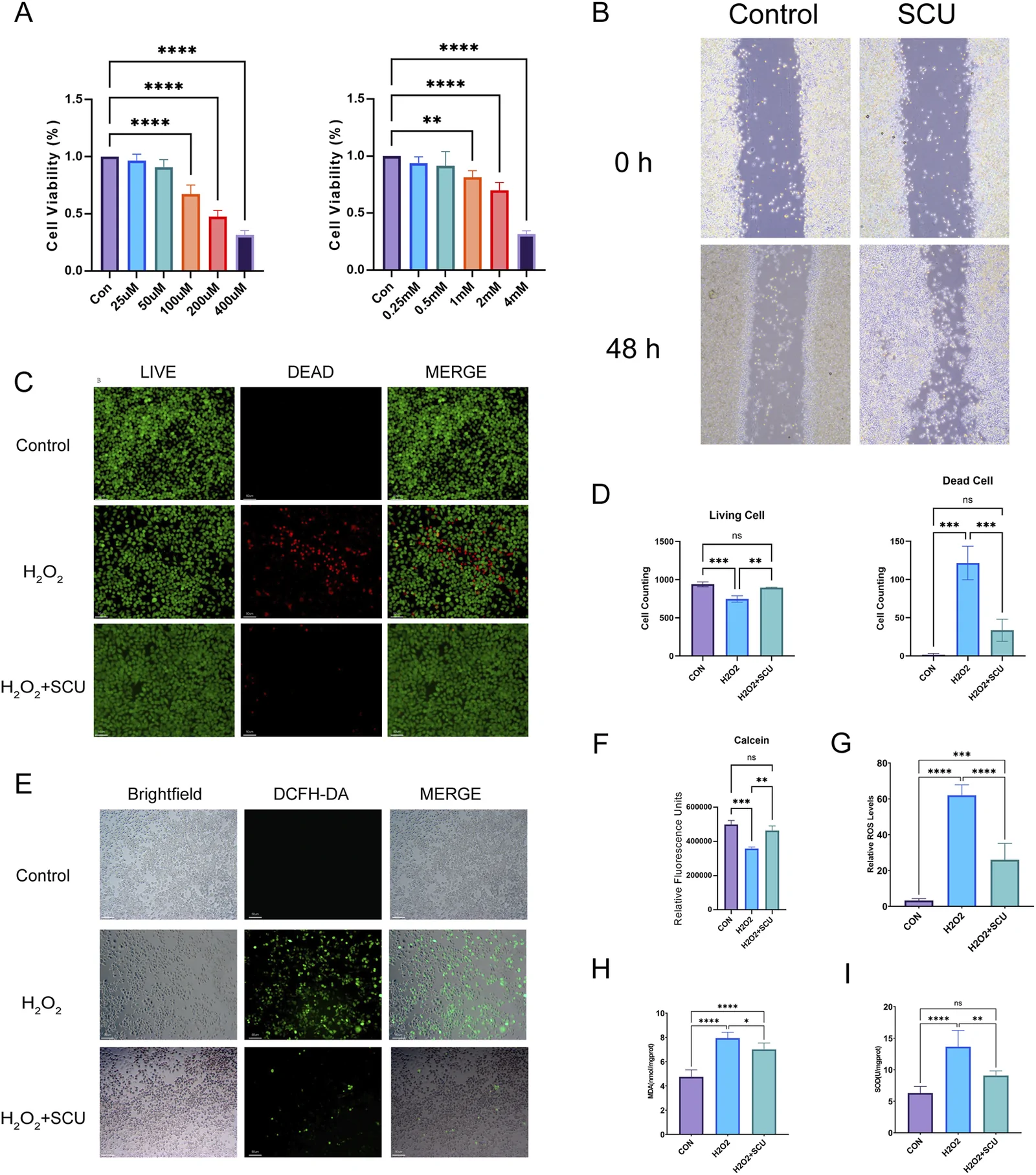
SCU enhances fibroblast scratch closure and attenuates H_2_O_2_-induced oxidative injury. CCK-8 assays were used to evaluate the effects of graded SCU concentrations (25-400 μ M; left, 24 h) and graded ${\text{H}}_{2}{\text{O}}_{2}$ concentrations (0.25-4 mM; right, 24 h) on fibroblast viability **(A)** Representative scratch-wound images of control and SCU-treated fibroblasts at 0 and 48 h are shown in **(B)** Representative Live/Dead staining images of control, ${\text{H}}_{2}{\text{O}}_{2}$ -injured, and ${\text{H}}_{2}{\text{O}}_{2}$ +SCU-treated fibroblasts are shown in **(C)** with quantification of live and dead cells in **(D)** Intracellular ROS levels were measured using DCFH-DA staining **(E)** and quantified in **(G)**. Relative Calcein fluorescence intensity in viable cells is shown in **(F)** MDA content and SOD activity were quantified in **(H,I)** respectively. One-way ANOVA with Tukey’s *post hoc* test was used for between-group comparisons. Data are presented as mean ± SEM from at least three independent experiments. ns, not significant; *p<0.05 , **p<0.01 , ***p<0.001 , ****p<0.0001 .

In the scratch-wound assay (Figure 3B), which included only the SCU-alone versus control condition (no ${\text{H}}_{2}{\text{O}}_{2}$ -injury arm), fibroblasts exposed to SCU alone showed greater gap closure at 48 h than untreated controls. Because no mitotic inhibitor was used, this assay is best interpreted as scratch closure reflecting migration with a possible proliferative contribution, and it reports only the baseline effect of SCU on uninjured fibroblasts.

Live/Dead staining (Figure 3C) showed a shift toward cell death after ${\text{H}}_{2}{\text{O}}_{2}$ exposure, and quantification (Figure 3D), together with relative Calcein signal in viable cells (Figure 3F), confirmed reduced survival in the ${\text{H}}_{2}{\text{O}}_{2}$ group. SCU co-treatment partially reversed these changes, indicating preservation of fibroblast viability under oxidative challenge.

DCFH-DA staining (Figure 3E) showed ROS accumulation in the ${\text{H}}_{2}{\text{O}}_{2}$ group, and quantitative analysis (Figure 3G) confirmed that SCU lowered intracellular ROS levels. ${\text{H}}_{2}{\text{O}}_{2}$ also increased MDA (Figure 3H) and altered SOD activity (Figure 3I), and both changes were attenuated by SCU. Thus, Figure 3 indicates that SCU increases scratch closure, limits oxidative cell death, suppresses ROS and lipid peroxidation, and restores antioxidant balance.

### 3.4 Scutellarein preserves mitochondrial structure and function in injured fibroblasts

Because oxidative damage often converges on mitochondria, we examined whether SCU preserved mitochondrial integrity in ${\text{H}}_{2}{\text{O}}_{2}$ -injured fibroblasts. MitoTracker Red imaging (Figure 4A) showed that control fibroblasts contained a relatively elongated mitochondrial network, whereas ${\text{H}}_{2}{\text{O}}_{2}$ exposure produced a fragmented, punctate pattern. Quantification of mean aspect ratio (Figure 4B) and mitochondrial count (Figure 4C) confirmed that ${\text{H}}_{2}{\text{O}}_{2}$ reduced mitochondrial elongation and increased fragmentation; SCU partially reversed both changes.

**FIGURE 4:**
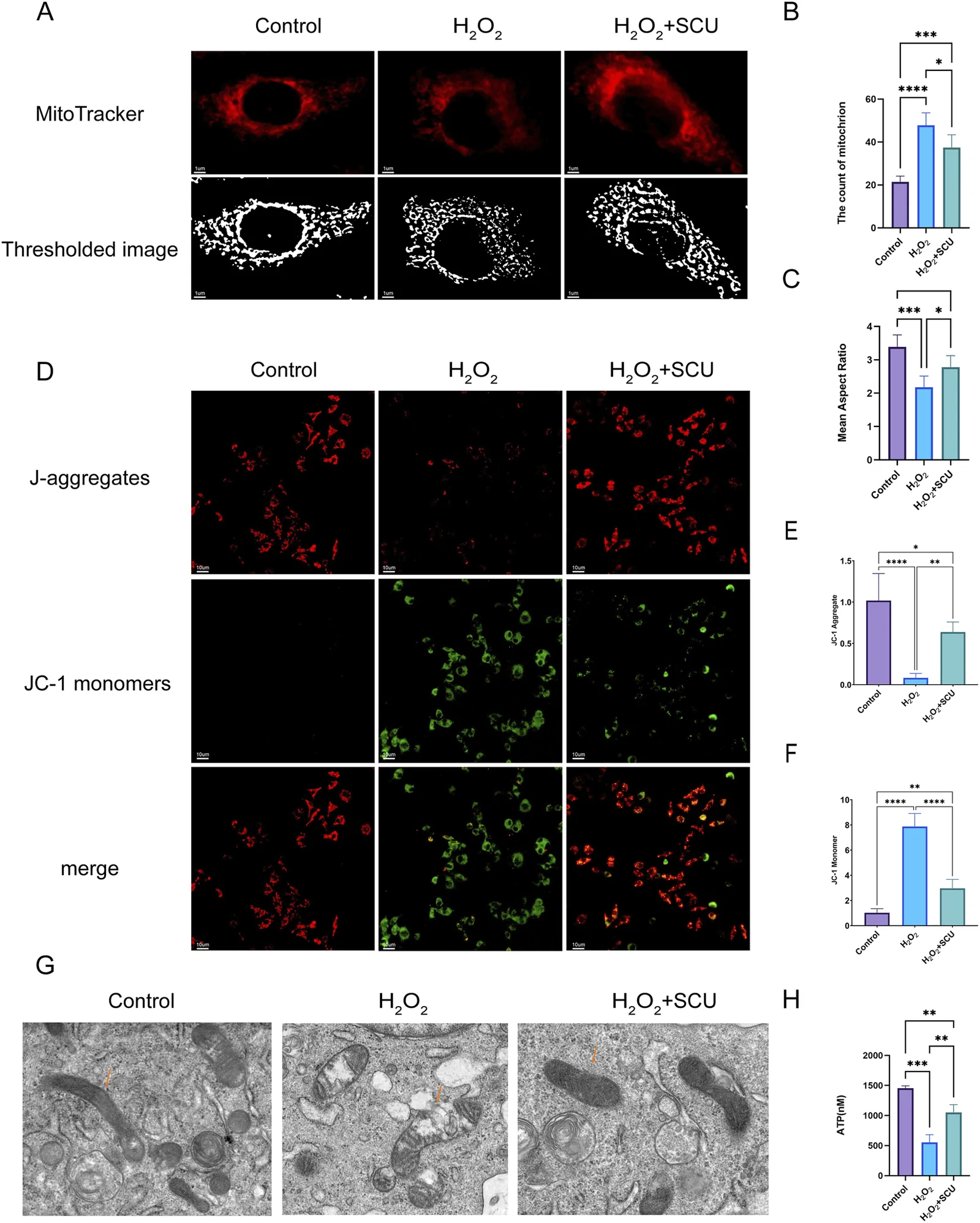
SCU preserves mitochondrial structure and function in injured fibroblasts. Representative MitoTracker Red images and corresponding thresholded images used for mitochondrial network analysis are shown in **(A)**. Mean aspect ratio and mitochondrial count were quantified in **(B,C)**. Respectively. Representative JC-1 staining images are shown in **(D)**. Red fluorescence indicates J-aggregates and green fluorescence indicates JC-1 monomers. Relative J-aggregate and monomer fluorescence intensities were quantified in **(E,F)**. Representative transmission electron microscopy images of mitochondrial ultrastructure are shown in **(G)**. Orange arrows indicating structural injury such as swelling and cristae disruption. Cellular ATP levels were quantified in **(H)**. Scale bars, 1 μ m in MitoTracker panels, 10 μ m in JC-1 fluorescence panels, and 500 nm in TEM panels. Data are from at least three independent experiments. One-way ANOVA with Tukey’s *post hoc* test was used for between-group comparisons. Data are presented as mean ± SEM. ns, not significant; *p<0.05 , **p<0.01 , ***p<0.001 , ****p<0.0001 .

JC-1 staining (Figure 4D) showed that ${\text{H}}_{2}{\text{O}}_{2}$ decreased red J-aggregate signal and increased green monomer signal, consistent with mitochondrial depolarization. Quantitative analysis (Figures 4E,F) confirmed loss of membrane potential in injured fibroblasts and partial restoration after SCU treatment.

TEM (Figure 4G) showed swollen mitochondria and cristae disruption after ${\text{H}}_{2}{\text{O}}_{2}$ exposure, whereas SCU-treated cells retained more preserved mitochondrial morphology. ATP measurement (Figure 4H) showed that these structural and electrochemical changes were accompanied by increased cellular ATP levels after SCU treatment.

### 3.5 Transcriptomics identify *P2rx1* as a candidate within calcium-mitochondrial pathways associated with SCU treatment

We next examined whether the cellular effects of SCU were accompanied by a coherent transcriptional program. Principal component analysis (Figure 5A) showed separation between the injury and SCU-treatment groups, indicating a transcriptomic shift. The volcano plot (Figure 5B) highlighted a differential-expression pattern in which *P2rx1* was significantly downregulated after SCU treatment, making it a candidate gene for further mechanistic analysis.

**FIGURE 5:**
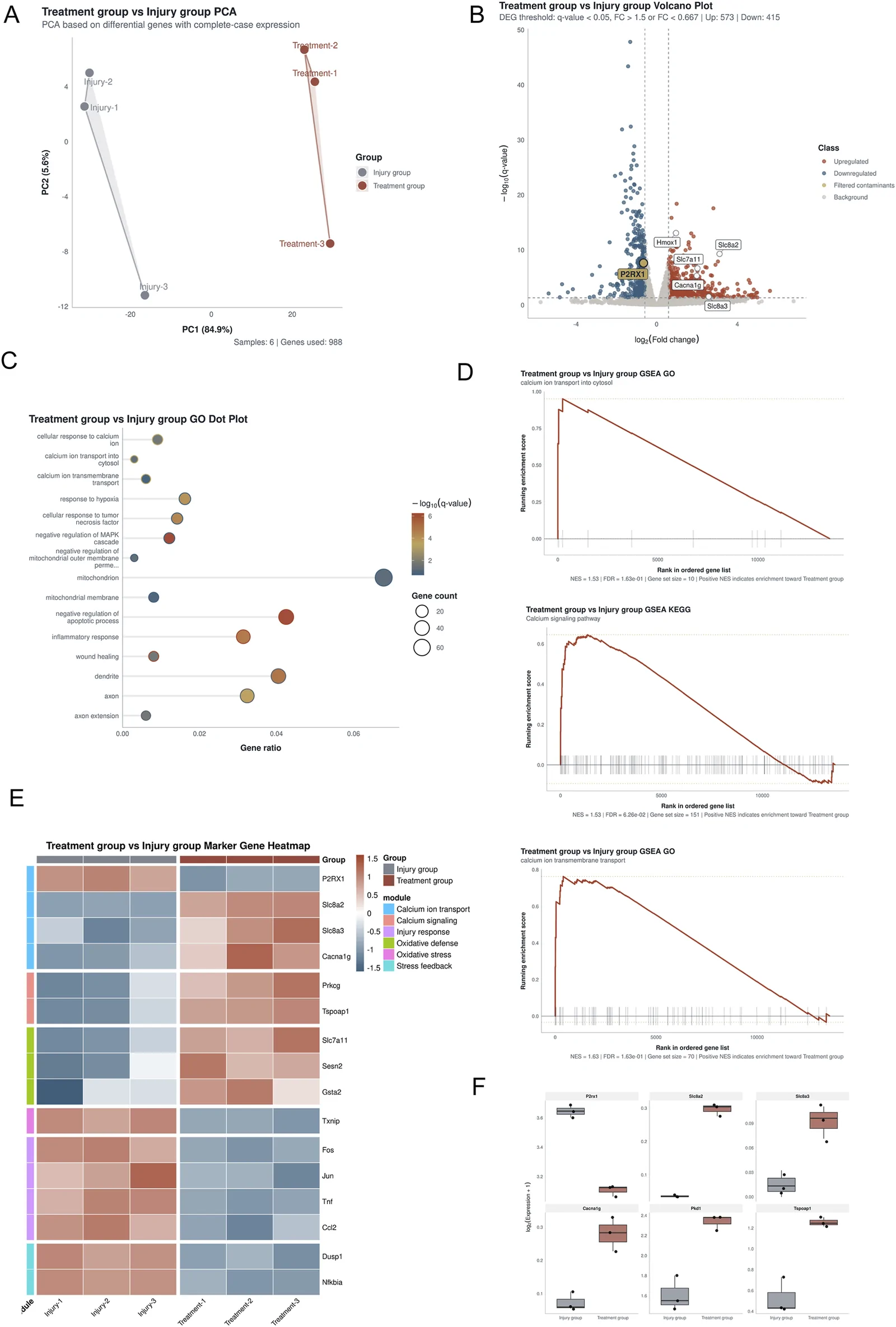
Transcriptomics identify *P2rx1* within calcium-stress pathways associated with SCU treatment. RNA-seq was performed on L929 fibroblast RNA ( ${\text{H}}_{2}{\text{O}}_{2}$ -injured vehicle group vs. SCU-treated group). Principal component analysis is shown in **(A)** n=3 per group. A volcano plot of differentially expressed genes is shown in **(B)** highlighting downregulated and upregulated genes in the treatment condition, including *P2rx1*. GO enrichment analysis is shown in **(C)** identifying pathways related to calcium transport, mitochondrial biology, inflammation, hypoxia response, and wound healing. GSEA plots for calcium ion transport into cytosol and calcium ion transmembrane transport are shown in **(D)** these gene sets showed nominal (non-significant, FDR ≈ 0.16) enrichment and are interpreted as a directional trend only. A heatmap of pathway-linked genes is shown in **(E)** and representative expression summaries for *P2rx1*, *Slc8a2*, *Slc8a3*, *Cacna1g*, *Pkd1*, and *Tspoap1* are shown in **(F)** Differential expression was assessed with DESeq2 using thresholds of adjusted q<0.05 and fold change >1.5 or <0.667 ; n=3 per group.

GO enrichment (Figure 5C) identified enriched terms related to calcium transport, mitochondrial pathways, inflammation-related biology, and wound-healing processes. GSEA (Figure 5D) indicated nominal, non-significant enrichment (FDR ≈ 0.16) of “calcium ion transport into cytosol” and “calcium ion transmembrane transport,” which we interpret only as a directional trend consistent with the calcium-mitochondrial hypothesis rather than as statistically significant evidence of calcium-pathway activation. These pathway-level findings link the tissue and cellular phenotypes observed in Figures 1–4 to a candidate calcium-mitochondrial regulatory axis.

Heatmap clustering and representative gene-expression summaries (Figures 5E,F) were consistent with this interpretation. SCU suppressed injury/stress-associated genes such as *Fos*, *Jun*, and *Tnf* while increasing oxidative-defense genes such as *Slc7a11* and *Gsta2*. Consistent with the calcium-signaling enrichment, Figure 5F displays representative calcium-transport/channel-related and membrane-associated genes, including *P2rx1*, *Slc8a2*, *Slc8a3*, *Cacna1g*, *Pkd1*, and *Tspoap1*. Thus, Figure 5 indicates that SCU treatment is associated with transcriptional changes involving calcium dysregulation, inflammatory signaling, oxidative defense, and wound-relevant biology.

### 3.6 ATP reversal implicates broad purinergic calcium signaling in the SCU response

At the functional level, calcium imaging showed that ${\text{H}}_{2}{\text{O}}_{2}$ induced intracellular calcium overload in L929 fibroblasts and that SCU suppressed this increase (Figures 6B,C). ATP addition counteracted the calcium-lowering effect associated with SCU treatment, supporting a functional link between purinergic calcium influx and the SCU response. Because ATP is a non-selective purinergic agonist that activates multiple P2X and P2Y subtypes expressed by fibroblasts, this reversal is interpreted as evidence for broad purinergic calcium influx rather than proof of P2RX1-specific signaling; P2RX1 remains a candidate inferred from its transcriptional downregulation.

**FIGURE 6:**
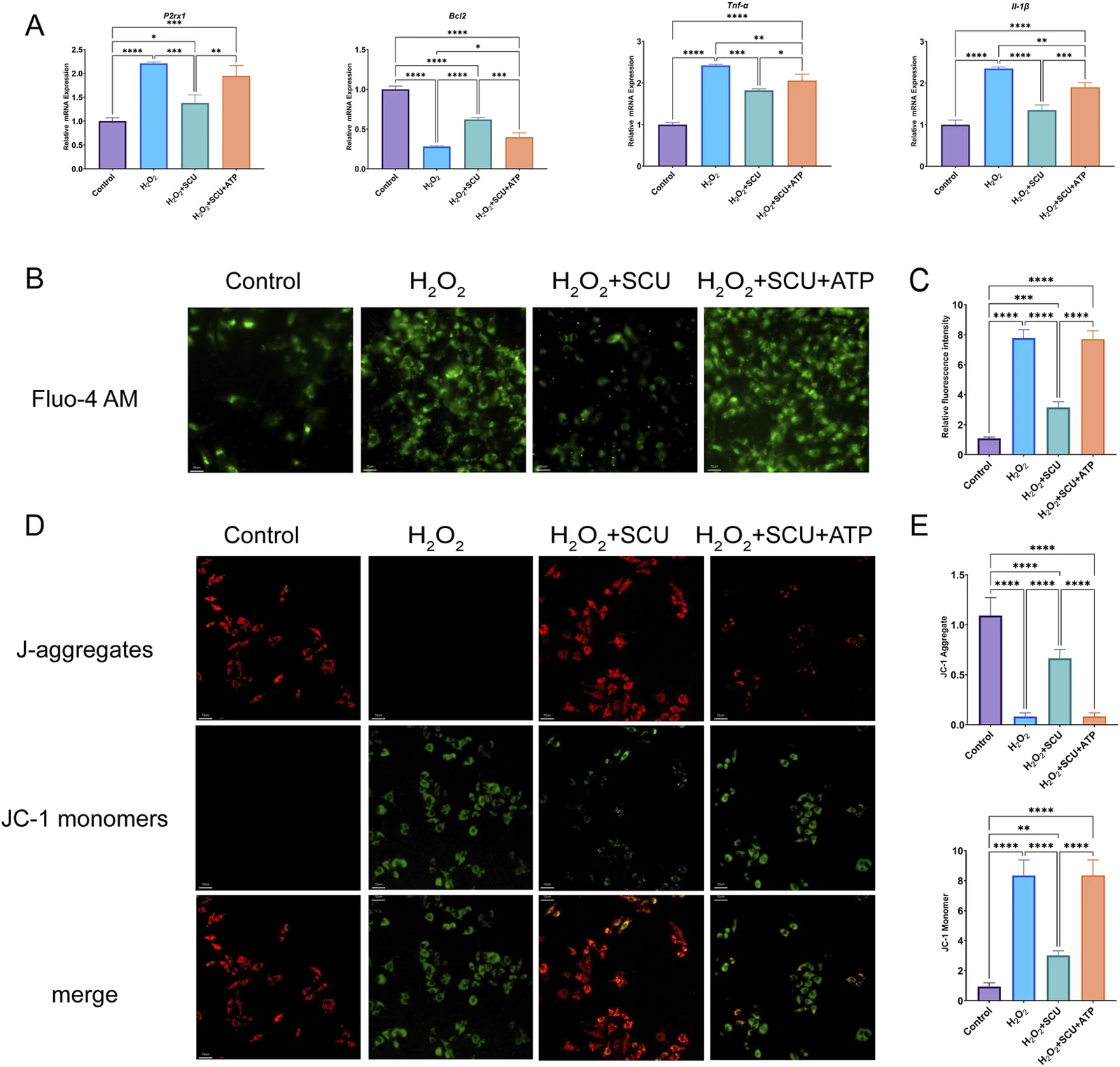
ATP reversal suggests involvement of broad purinergic calcium signaling in the SCU response. RT-qPCR analysis of *P2rx1*, *Bcl2*, *Il1b*, and *Tnf* expression in L929 fibroblasts across the indicated treatment groups, including the ${\text{H}}_{2}{\text{O}}_{2}$ +SCU + ATP reversal group, is shown in **(A)**. Representative intracellular calcium fluorescence images are shown in **(B)**. Quantification in **(C)**. Representative JC-1 staining images are shown in **(D)**. Quantification of J-aggregate and monomer fluorescence in **(E)**. Data are from at least three independent experiments. One-way ANOVA with Tukey’s *post hoc* test was used for between-group comparisons. Data are presented as mean ± SEM. ns, not significant; *p<0.05 , **p<0.01 , ***p<0.001 , ****p<0.0001 .

Downstream readouts showed a similar pattern. In the same reversal framework, ATP addition counteracted the SCU-mediated preservation of mitochondrial membrane potential (Figures 6D,E), indicating that calcium overload is functionally linked to mitochondrial dysfunction in this system. RT-qPCR readouts from L929 fibroblasts (Figure 6A) showed that ATP also blunted the SCU-mediated increase in *Bcl2* and restored higher expression of *Tnf* and *Il1b*. Collectively, Figure 6 is consistent with a model in which SCU-mediated fibroblast protection involves reduced purinergic calcium overload and preservation of mitochondrial homeostasis, with P2RX1 emerging as a candidate purinergic signal associated with the response rather than a confirmed direct target.

## 4 Discussion

In this study, SCU promoted diabetic wound repair at both tissue and cellular levels. *In vivo*, SCU accelerated wound closure and enhanced re-epithelialization, Masson-positive collagen deposition, proliferative α -SMA-positive wound-cell recovery, and angiogenesis. These phenotypes align with the established concept that successful diabetic wound healing depends on restoration of fibroblast activity, matrix remodeling, vascular support, and coordinated progression beyond the chronically inflamed wound state (Pawar et al., 2021; Wan et al., 2021; Rai et al., 2023; Yan et al., 2025). The data therefore indicate that SCU promoted multiple linked dimensions of reparative wound biology in this experimental model rather than affecting wound closure alone. To our knowledge, this is the first study to associate SCU treatment with preservation of calcium-mitochondrial homeostasis and with P2RX1-related purinergic signaling in diabetic wound repair, extending the previously documented antioxidant and anti-inflammatory actions of scutellarein (Pandith et al., 2013; Nazaruk and Galicka, 2014; Lai and Li, 2024).

The present work also provides a fibroblast-centered assessment of SCU action. *In vitro*, SCU enhanced scratch closure and protected cells from ${\text{H}}_{2}{\text{O}}_{2}$ -induced death while reducing ROS and MDA and restoring antioxidant balance. These findings are consistent with evidence that impaired cell movement, reduced survival, and oxidative injury are major barriers to diabetic wound repair (Song et al., 2025; Elajaili et al., 2025; Tian et al., 2025). They also align with reports that antioxidant or redox-correcting interventions can restore fibroblast-mediated repair programs and improve wound quality (Yan et al., 2025; Wlaschek and Scharffetter-Kochanek, 2005; Wei et al., 2009). Although prior SCU-related literature has not focused on diabetic skin wounds, previous reports of anti-inflammatory and fibroblast-relevant effects support the biological plausibility of these observations (Pandith et al., 2013; Nazaruk and Galicka, 2014; Miao et al., 2020).

Beyond bulk oxidative-stress readouts, the mitochondrial data provide an additional mechanistic layer. SCU reduced mitochondrial fragmentation, preserved membrane potential, improved ultrastructural integrity, and restored ATP production in oxidatively injured fibroblasts. These findings are consistent with literature linking redox imbalance to mitochondrial collapse and impaired fibroblast or tissue-repair function (Ma et al., 2009; Wu et al., 2010; Zhou et al., 2022). They also agree with recent wound-oriented studies showing that interventions that correct oxidative or metabolic stress can improve fibroblast performance and diabetic wound repair (Zhou et al., 2022; Yu et al., 2022; Zhang et al., 2025; Guo et al., 2025).

The most consistent mechanistic finding in this study emerged from the convergence of transcriptomics and ATP-reversal experiments. RNA-seq showed transcriptional separation between injured and SCU-treated fibroblasts, identified *P2rx1* as a significantly downregulated gene, and linked the treatment response to calcium transport/signaling, mitochondrial biology, inflammation, and wound-healing pathways. These observations are consistent with transcriptomic evidence that wound-repair outcomes are shaped by coordinated stromal, inflammatory, and metabolic programs (Theocharidis et al., 2020). Importantly, the RNA-seq signal was not confined to *P2rx1*: SCU also modulated a broader calcium-handling and membrane-associated gene set, including the sodium/calcium exchangers *Slc8a2* and *Slc8a3*, the T-type calcium channel *Cacna1g*, and *Pkd1* (which encodes polycystin-1, a mechanosensitive, calcium-associated membrane protein), together with oxidative-defense genes (*Slc7a11*, *Gsta2*) and stress/inflammatory genes (*Fos*, *Jun*, *Tnf*). This multi-gene pattern points to a coordinated program of calcium-homeostasis restoration and redox recovery rather than a single-gene mechanism, and it reinforces the view that the calcium-mitochondrial axis, rather than P2RX1 alone, is central to the SCU response. The data are also consistent with prior evidence that P2RX1 is a calcium-conducting purinergic receptor whose activity influences calcium-dependent physiology and inflammatory signaling (Lalo et al., 2010; Qiang et al., 2025; Maitre et al., 2015; Wang et al., 2017). Notably, P2RX1 modulation has been reported to preserve mitochondrial dynamics and alleviate tissue injury *in vivo* (Zhuang et al., 2021), supporting P2RX1 as a candidate purinergic signal, not a validated target, in the SCU response. In the ATP-reversal experiments, purinergic stimulation counteracted the SCU-associated improvement in calcium overload, mitochondrial membrane potential, and inflammatory/survival gene expression. Together, these data support involvement of broad purinergic calcium-mitochondrial signaling and place P2RX1 as a plausible candidate mediator, while not establishing direct receptor causality.

Scutellarein has also been reported to inhibit fibroblast differentiation and proliferation in bleomycin-induced pulmonary fibrosis (Miao et al., 2020). This is not in conflict with our pro-survival observations: in pulmonary fibrosis the fibroblast compartment is pathologically over-activated, whereas in the diabetic wound it is oxidatively stressed, dysfunctional, and insufficiently proliferative. Scutellarein therefore appears to exert context-dependent, homeostatic effects-restraining pathological fibrotic activation while preserving the survival and function of oxidatively injured fibroblasts-a dual behaviour consistent with the redox-regulating actions of many flavonoids. In the present study, P2RX1 is discussed strictly as a candidate purinergic signal implicated by its transcriptional downregulation and the functional ATP-reversal experiments, rather than a validated direct target. Definitive attribution of a direct ligand-receptor interaction would require biophysical binding assays (such as the cellular thermal shift assay [CETSA], surface plasmon resonance [SPR], or drug affinity responsive target stability [DARTS]), together with receptor-specific genetic or pharmacological perturbations (e.g., *P2rx1* siRNA or the selective antagonist NF449).

From a translational perspective, the pharmacological and pharmacokinetic profile of scutellarein should be considered when interpreting the present findings. Published pharmacological and pharmacokinetic studies indicate that scutellarein exerts broad biological activities and can inhibit uridine 5’-diphosphate-glucuronosyltransferases and cytochrome P450 enzymes, which may have safety implications, particularly for drug-interaction risk (Lai and Li, 2024). As with many flavonoids, scutellarein is characterized by limited oral bioavailability and extensive conjugation metabolism, which constrains systemic administration and supports the rationale for local topical delivery to the wound bed. In the present study, repeated local administration of a 200 μ M SCU solution accelerated closure of diabetic wounds in mice; however, local drug exposure, penetration depth, retention, and systemic absorption were not quantified. Future translational development should therefore evaluate formulation-based delivery strategies (e.g., hydrogels, nanoemulsions, or dressing-based systems) that improve solubility, stability, and retention at the wound site, and should establish dose-exposure, pharmacokinetic, and safety profiles in wound-relevant settings and ultimately in clinical studies. These considerations underscore the exploratory, preclinical nature of the present findings and the need for further validation before any clinical application is contemplated.

Several limitations should be acknowledged. First, the mechanistic conclusions rely on transcriptomics and ATP reversal rather than direct receptor perturbation; P2RX1 was not silenced, overexpressed, or pharmacologically antagonized, and ATP is a broad purinergic stimulus that does not isolate P2RX1-specific signaling, so the receptor-specific attribution remains untested and requires NF449 and/or *P2rx1* siRNA experiments. Second, the *in vitro* work used the immortalized L929 line, whose P2RX1 expression and function were not directly measured and which may differ from primary dermal fibroblasts; validation in primary dermal fibroblasts, including diabetic donor-derived cells, is needed. Third, the ${\text{H}}_{2}{\text{O}}_{2}$ model recapitulates acute oxidative injury but not the chronic hyperglycemic milieu, and a high-glucose or high-glucose-plus- ${\text{H}}_{2}{\text{O}}_{2}$ model was not tested, representing a future direction. Fourth, the scratch-wound assay lacked a mitotic inhibitor, so closure reflects migration with a possible proliferative contribution, and it reports only baseline (non-injured) effects. Fifth, effects of SCU on normoglycemic (non-diabetic) wound healing were not assessed; although SCU-alone conditions in the *in vitro* assays showed no reduction in fibroblast viability at the tested concentrations, formal evaluation of the specificity and safety of SCU in normal healing is warranted. Sixth, SCU was administered locally at a single repeated dose (200 μ M), and local tissue concentration, penetration, and pharmacokinetic relationships were not characterized. Seventh, only male mice were used in the *in vivo* model; therefore, potential sex-dependent effects of SCU on diabetic wound repair were not evaluated, and studies in female diabetic mice are planned. Despite these limitations, the overall dataset indicates that SCU promoted diabetic wound healing and fibroblast resilience in experimental models, with evidence implicating purinergic/P2RX1-associated calcium stress and oxidative mitochondrial injury.

## 5 Conclusion

In experimental models, SCU promoted diabetic wound repair and protected fibroblasts from oxidative injury while improving redox balance, preserving mitochondrial homeostasis, and reducing purinergic calcium overload. These findings support calcium-mitochondrial homeostasis as a mechanistically relevant axis in SCU-mediated fibroblast protection. Broad purinergic signaling, possibly involving P2RX1, may contribute to these effects; direct receptor-perturbation studies (e.g., the P2X1-selective antagonist NF449, or *P2rx1* knockdown/overexpression) are required to establish the specific contribution of P2RX1.

## Data Availability

The datasets presented in this study can be found in online repositories. The names of the repository/repositories and accession number(s) can be found in the article/Supplementary Material. The RNA-seq data are available in GEO under accession number GSE333609.

## Supplementary material

SUPPLEMENTARY FIGURE 1 Chemical structure of scutellarein. Scutellarein (5,6,7,4’-tetrahydroxyflavone; CAS 529-53-3; molecular formula ${\text{C}}_{15}{\text{H}}_{10}{\text{O}}_{6}$ ; exact mass 286.05 g/mol), the plant-derived flavonoid metabolite investigated in this study, is shown.
